# Cystatin C as a Marker for Glomerular Filtration Rate in Critically Ill Neonates and Children: Validation Against Iohexol Plasma Clearance

**DOI:** 10.1016/j.ekir.2023.05.028

**Published:** 2023-06-03

**Authors:** Nori J.L. Smeets, A. Bökenkamp, Anders Grubb, Saskia N. de Wildt, Michiel F. Schreuder

**Affiliations:** 1 Department of Pharmacology and Toxicology, Radboud University Medical Center, Radboud Institute for Health Sciences, Nijmegen, The Netherlands; 2 Intensive Care and Department of Pediatric Surgery, Erasmus MC Sophia Children’s Hospital, Rotterdam, The Netherlands; 3 Department of Pediatric Nephrology, Emma Children’s Hospital, Amsterdam University Medical Centers, Amsterdam, The Netherlands; 4 Department of Clinical Chemistry and Pharmacology, Laboratory Medicine, Lund University, Lund, Sweden; 5 Division of Pediatric Nephrology, Department of Pediatrics, Radboud University Medical Center, Amalia Children’s Hospital, Nijmegen, The Netherlands

## Introduction

Accurately measuring glomerular filtration rate (GFR) is important to ensure the timely detection of alterations in GFR and to adapt pharmacotherapy and fluid therapy accordingly. In nonstable situations, GFR assessment by the most widely used marker, serum creatinine (SCr) is hampered by its long half-life because of a large volume of distribution (reflecting total body water) and systemic errors from muscle mass. In addition, in neonates, SCr-based GFR assessment is complicated by the influence of maternal creatinine because creatinine crosses the placenta.^1^ Because of these limitations, cystatin C (cysC) has been proposed as an alternative marker of GFR. CysC has consistent stable plasma levels from 1 year of age and its levels are independent of muscle mass.^2^ In addition, cysC is distributed in the extracellular volume only, explaining the shorter half-life compared to creatinine. Therefore, the value of cysC as a marker for GFR in the neonatal and pediatric population has been investigated by many and is considered promising.

However, in neonates, the value of cysC as a marker for GFR has never been investigated with gold standard measured GFR (mGFR). In a large cohort of children with chronic kidney disease, age-dependent and sex-dependent clinical cysC equations to estimate GFR were developed, indicating nonsignificant bias and high accuracy of these equations when compared to iohexol-based mGFR.^3^ Considering that estimated GFR (eGFR) equations were developed and validated in a homogenous population with a median age of 13 (interquartile range [IQR] 9–16) years, the value of cysC remains uncertain in patients without chronic kidney disease and patients of young age. Therefore, the added value of cysC for GFR determination in critically ill neonates and children remains debatable. In critically ill adults, cysC outperformed SCr for the detection of impaired GFR.^4^ However, because cysC levels are significantly affected by thyroid disorders^5^ and corticosteroids,^6^ using cysC-based eGFR equations in the critically ill warrants caution. Due the drawbacks of SCr-based eGFR equations, and the importance of accurate GFR determination, we aimed to test our hypothesis that cysC-outperforms SCr as a marker for GFR in critically ill term-born neonates and children.

## Results

**Figure 1:**
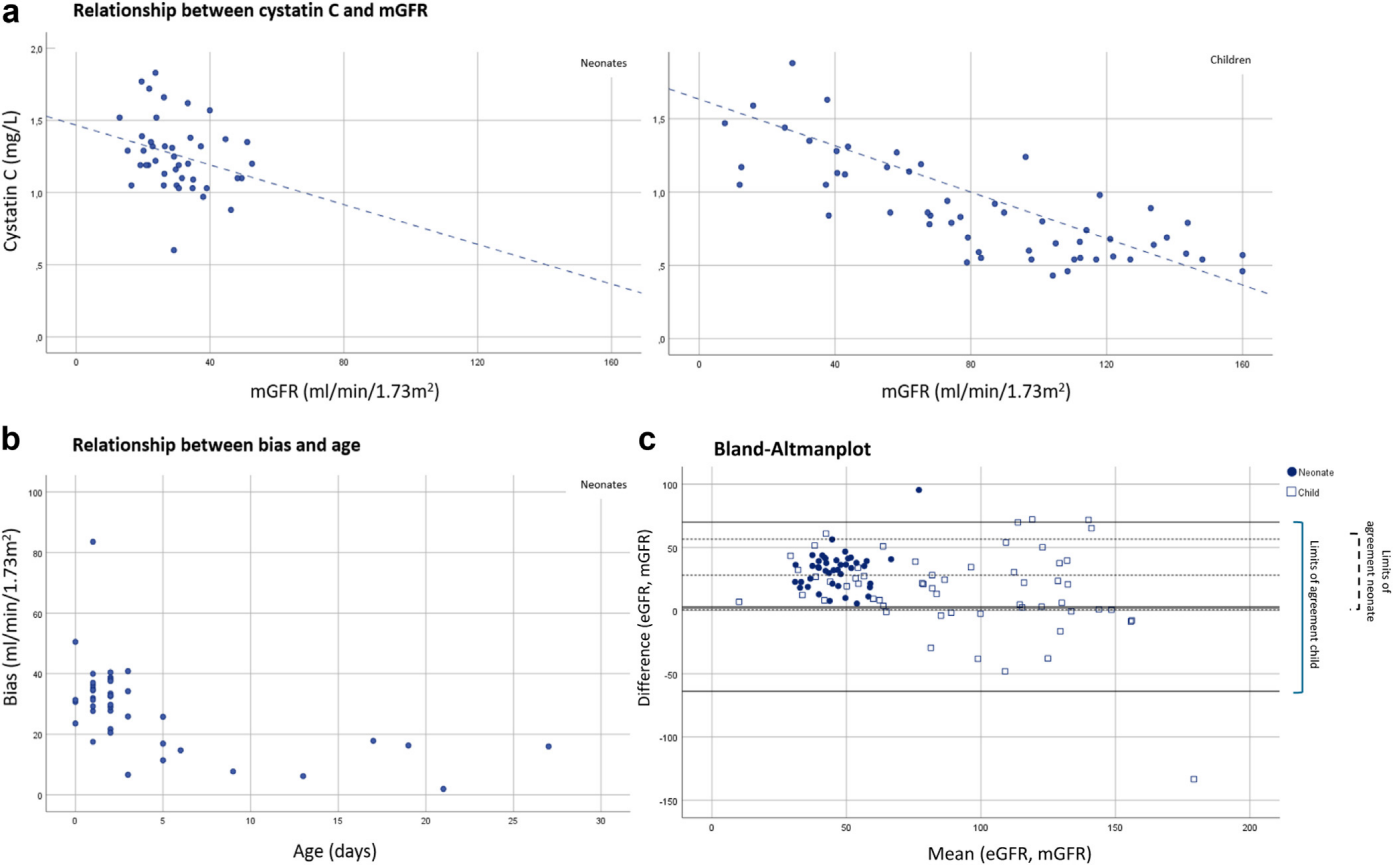
Correlation between cystatin C levels and iohexol-based mGFR in neonates (upper left panel) and children (upper right panel). Dashed line represents trend line fitted to the data by using linear regression. For visualization purposes, we excluded 2 patients (children) from the figures with the following values: mGFR 245.9 ml/min/1.73 m^2^; cysC 0.7 mg/l and mGFR 6.6 ml/min/1.73 m^2^; cysC 5.5 mg/l. (b) The relationship between bias and age in neonates for the Schwartz equation. (c) Bland-Altman plot demonstrating the agreement between eGFR according to the Pierce equation (age-dependent k-value) and mGFR. For neonates, mean difference was 28.6 ml/min/1.73 m^2^ with limits of agreement between 0.6–56.6 ml/min/1.73 m^2^. For children, mean difference was 3.1 (limits of agreement −63.9 to 70). mGFR; measured glomerular filtration rate.

Measurement of cysC or iohexol-based mGFR failed in 6 patient; demographic data in these patients did not systematically differ from the analyzed cohort. At inclusion, median age was 2 (range 0–27) days for neonates (*n* = 42) and 6.0 (range 0.1–17.2) years for children (*n* = 57). Median cysC levels were 1.2 (IQR 1.1–1.4) mg/l for neonates, and 0.8 (IQR 0.6–1.2) mg/l for children. Neonates had a median mGFR of 29.2 (IQR 22.3–35.5) ml/min per 1.73 m^2^, and children had a mediam mGFR of 82.2 (IQR 43.3–115.5) ml/min per 1.73 m^2^. According to the Kidney Disease Improving Global Outcomes criteria, 26 patients were diagnosed with acute kidney injury (8 neonates, 18 children). In neonates, the relationship between cysC and mGFR was statistically significant, but poor (*r* = −0.30 [95% confidence interval −0.55 to 0.00], *P* = 0.050), whereas in children, this relationship was stronger (*r* = −0.77 (95% CI −0.86 to −0.62), *P* < 0.001) (Figure 1a).

Correspondingly, equations to estimate GFR solely based on cysC, did not perform well in neonates with significant overestimation of mGFR by eGFR (34.1–43.7 ml/min per 1.73 m^2^) and unacceptably poor accuracy (Table 1). Performance was higher in children, even though all included eGFR equations significantly overestimated GFR (19.3–37.0 ml/min per 1.73 m^2^). Of all cysC-based eGFR equations, eGFR Schwartz and Pierce yielded the highest accuracy and agreement with mGFR. Visualization of the relationship between bias and age in neonates indicates that bias is age-dependent and highest in neonates below the age of 5 days (Figure 1b). Combining cysC and SCr in the extensive Schwartz equation increased the performance of eGFR calculations compared to solely cysC-based equations. Yet, agreement was not as high and bias not as low when compared to eGFR equations based on SCr alone. Performance of SCr-based GFR equations in the same cohort demonstrated accuracy up to 67 % and bias as low as 0.0 (−6.4 to 5.2) ml/min per 1.73 m^2^.^7^ It should be noted, however, that none of our equations were established in children under the age of 2 years, which may account for some of the inaccuracy compared to other studies.^3^^,^^8^

**Table 1:** Agreement between mGFR and multiple eGFR equations

Method of GFR determination	Median GFR (IQR) In ml/min per 1.73 m^2^	Median difference between eGFR and mGFR (IQR) in ml/min per 1.73 m^2^	*P*-value^a^	Accuracy (%)^b^
TOTAL				
mGFR	42.9 (26.5–96.1)			
eGFR Pierce-age and sex dependent	70.2 (59.3–112.5)	25.3 (8.3–38.8)	<0.001	28.3
eGFR Pierce-constant	72.5 (59.9–108.6)	28.2 (12.2–40.9)	<0.001	29.3
eGFR Schwartz	74.9 (63.1–107.4)	29.3 (13.8–43.8)	<0.001	26.3
eGFR Schwartz (combined equation)	63.5 (37.1–101.1)	10.8 (0.7–20.0)	<0.001	44.4
eGFR FAS	80.7 (67.2–118.9)	36.7 (19.9–49.6)	<0.001	24.2
eGFR Zappitelli	82.5 (66.5–129.8)	41.1 (21.3–55.3)	<0.001	18.2
eGFR CAPA	110.4 (89.6–147.2)	61.7 (37.5–79.4)	<0.001	16.2
eGFR Pierce (creatinine based)	59.7 (29.5–105.2)	3.9 (–6.0 to 20.5)	0.002	59.4
Children				
mGFR	82.2 (43.3– 115.5)			
eGFR Pierce-age and sex dependent	95.0 (66.3–134.8)	20.8 (0.8–34.2)	<0.001	42.1
eGFR Pierce-constant	97.5 (70.0–138.7)	19.8 (−1.4 to 36.9)	<0.001	47.4
eGFR Schwartz	96.5 (71.0–133.6)	19.3 (2.1–35.0)	0.002	43.9
eGFR Schwartz (combined equation)	88.4 (67.1–119.1)	9.4 (−10.7 to 25.5)	0.030	52.6
eGFR FAS	106.0 (76.2–150.4)	31.3 (9.2–47.1)	<0.001	40.4
eGFR Zappitelli	113.5 (77.1–170.9)	37.0 (16.3–62.6)	<0.001	29.8
eGFR CAPA	132.9 (99.1–173.7)	52.4 (19.3–76.7)	<0.001	28.1
eGFR Pierce (creatinine based)	89.8 (60.9–127.9)	7.1 (−10.5 to 30.0)	0.0016	50.0
Neonates				
mGFR	29.2 (22.3–35.5)			
eGFR Pierce-age and sex dependent	61.8 (55.3–68.9)	34.1 (21.3–40.0)	<0.001	9.5
eGFR Pierce-constant	63.5 (55.2–72.5)	34.8 (24.4–44.8)	<0.001	4.8
eGFR Schwartz	68.0 (60.5–74.4)	39.9 (26.1–45.7)	<0.001	2.4
eGFR Schwartz (combined equation)	38.1 (31.5–48.8)	12.1 (5.4–15.8)	<0.001	33.3
eGFR FAS	72.7 (64.1–80.2)	43.7 (30.0–52.5)	<0.001	2.4
eGFR Zappitelli	73.0 (63.0–81.8)	43.0 (28.6–53.0)	<0.001	2.4
eGFR CAPA	99.0 (85.7–110.7)	66.7 (51.9–80.2)	<0.001	0.0
eGFR Smeets (creatinine based)	26.3 (18.9–36.5)	0.0 (−6.4 to 5.2)	0.847	74.4

CAPA, Caucasian and Asian pediatric and adult subjects; eGFR, estimated glomerular filtration rate; FAS, full age spectrum; mGFR, measured glomerular filtration rate.

Median GFR and median bias with corresponding IQR are displayed in ml/min/1.73.

a Comparison of mGFR and particular eGFR using the Wilcoxon signed rank test.

b Percentage of patients of whom eGFR was within ±30% of mGFR.

Concluding, our results indicate a significant difference in cysC and mGFR correlation between critically ill neonates and children. In neonates, whereas cysC values and mGFR were borderline related, performance of cysC-based eGFR equations was disappointing with unacceptable accuracy and high bias. These results question the value of cysC as a filtration marker in neonates, especially in those <5 days of age. In critically ill children, the correlation was better and cysC could therefore be of value in in-patients in whom the use of SCr is hampered (e.g., low muscle mass).

Only one other study investigated the correlation between cysC levels and mGFR in neonates, albeit in preterms. In neonates (gestational ages 28–34 weeks, postnatal ages 4–7 days; *n* = 20), cysC was correlated with inulin-based mGFR, (*r* = 0.766, *P* < 0.001).^9^ These neonates were older and more stable compared to our cohort. In critically ill neonates and children, cysC was only investigated against SCr-based GFR. In critically ill neonates (*n* = 135), cysC-based eGFR was not superior to SCr-based eGFR in diagnosing acute kidney injury.S1 In 2 cohorts of critically ill children, cysC was compared with creatinine clearance. In 25 critically ill children (mean age 3 [range 0–14] years), cysC was not correlated with creatinine clearance (*r* = 0.390, *P* = 0.054).S2 In 107 children aged 10 (IQR 3–36) months of age, the sensitivity to detect acute kidney injury was significantly higher using cysC than SCr.S3 However, the correlation between cysC and mGFR as well as the performance of cysC-based eGFR equations in critically ill neonates and children was never assessed. The observed difference in performance between neonates and children is likely because of an age-related effect on the relation between cysC and mGFR. First, GFR increases rapidly during the first days of age,S4 which, if compared to stable situations, likely decreases the correlation between cysC and mGFR. Although cysC does not cross the placenta, metabolic activity and tubular cell transport display extensive variation and maturation,S5 presumably limiting its accuracy for determining GFR in the neonatal period. Lastly, of note are the difficulties accompanying cysC measurements and assay diversity between measurement by particle-enhanced nephelometric immunoassay and particle-enhanced turbidimetric immunoassay. The latter yields up to 28% higher values.S6 To streamline cysC measurements and offer equivalence between laboratories, the use of the international calibrator proposed by the International Federation of Clinical Chemistry in 2012 is recommended. However, different methods remain in use, leading to suboptimal performance because of imprecision and nonequivalence between cysC results.

In conclusion, to the best of our knowledge, we are the first to report cysC levels in combination with mGFR in critically ill term-born neonates and children. We demonstrate that neonates significantly differ from children with regard to their cysC and iohexol-based mGFR correlation. Our results showed a poor correlation in neonates, whereas in children this was moderate. We believe that none of the currently available cysC-based equation should be recommended in the context of critically ill pediatric patients. Furthermore, future cysC studies should not consider the pediatric population as a whole, but rather need to address the influence of age on this GFR marker. In addition, improvement and harmonization of assay-specific equations is needed. Together, this may ensure the timely detection of GFR alterations and thereby enable GFR-adapted drug dosing in this vulnerable population.

## Disclosure

All the authors declared no competing interests.

## Supplementary Material

**Supplementary Methods.**

**Supplemental References.**
