# Supplementary material for: Cystatin C as a Marker for Glomerular Filtration Rate in Critically Ill Neonates and Children: Validation Against Iohexol Plasma Clearance
Source: Kidney Int Rep. 2023 Jun 3;8(8):1672–5. doi: 10.1016/j.ekir.2023.05.028 (PMC10403645; doi:10.1016/j.ekir.2023.05.028)
Supplement: Supplementary File (PDF) [file mmc1.pdf]

Supplementary material

Abbreviations

GFR	glomerular filtration rate
mGFR	measured glomerular filtration rate
cysC	cystatin C
PENIA	particle enhanced nephelometric immunoassay
eGFR	estimated glomerular filtration rate
JBM	Jødal and Brøchner–Mortensen
BSA	body surface area

Methods

The methods of this single-centre, prospective study and characteristics of included patients were previously described in detail [1]. In short, 105 critically ill term-born neonates and children aged 0-18 years with at least one failing organ and an indwelling central venous line were included after signed informed consent.

After inclusion, iohexol (Omnipaque[illegible] 300mg/mL, GE Healthcare, Chicago, Illinois, USA) was administered as a single bolus dose adapted to bodyweight as follows: <10kg, 1mL; 10–20 kg, 2mL; 20–30 kg, 3mL; 30–40 kg, 4mL; ≥40 kg, 5mL [2]. To determine measured glomerular filtration rate (mGFR), blood samples were drawn for analysis of iohexol concentrations at two, five and seven hours after administration. Two-point blood sampling at 2 and 5 hours after administration is a validated method for mGFR determination in children [2]. To enhance accuracy as low glomerular filtration rate (GFR) values were expected, we added another sampling point at 7 hours after administration for neonates with a bodyweight of at least 3.5kg and children older than 28 days of age. In neonates below the weight of 3.5kg, two-point blood sampling was used as three-point sampling would have exceeded the maximum amount of blood withdrawn. Cystatin C (cysC) levels were determined two hours after iohexol infusion for estimated glomerular filtration rate (eGFR) determination to reflect the clinical situation in which cysC is measured at point of care and to correspond to the first blood withdrawal point needed for mGFR, preventing an extra blood withdrawal.

CysC levels were measured by particle enhanced nephelometric immunoassay (PENIA) using an Atellica nephelometer (Siemens), as calibrated by the International Federation of Clinical Chemistry [3]. Iohexol plasma concentrations were determined at the Leiden University Medical Centre, Leiden, the Netherlands, using a validated high-performance liquid chromatography diode array detection assay [4]. The assay was validated according to the European Medicines Agency bioanalytical method validation guidelines [5].

mGFR based on iohexol plasma clearance was calculated based on the ratio between the administered iohexol dose and the area under the plasma concentration time curve. A slope-intercept method, using the Jødal and Brøchner–Mortensen (JBM) formula with early normalization to 1.73 m^2 body surface area (BSA) was employed as this method was previously validated in children with CKD [2, 6]. The Haycock-formula was used to calculate BSA [7 8].

Analyses were performed separately for neonates (<28 days of age) and children (>28 days of age). Spearman correlation coefficients were calculated to determine the correlation between cysC and mGFR. The following five cysC-based eGFR equations were used to estimate GFR: Pierce (with constant and age and sex dependent k) [9], Schwartz [10], Full Age Spectrum (FAS) [11], Zappitelli [12] and the Caucasian, Asian, Paediatric and Adult (CAPA) equation [13]. Last, the extensive Schwartz equation incorporating cysC, serum creatinine, blood urea nitrogen, height and sex was also included in our analysis [14]. To enable comparison to creatinine-based eGFR equations, the Pierce (creatinine based)[9] and Smeets[S4] equations were included.

There is known variability in reported results for measurement of cysC among and even within a measurement procedure. Consequently, the calculated eGFR values using these cystatin C measured concentrations also show variability. To minimize this variability, an international reference material was prepared in 2010 by the International Federation for Clinical Chemistry and Laboratory Medicine Working Group on Standardization of Cystatin C [13]. To enable comparison across the different cysC-based eGFR equations, cysC values were corrected for equations that were developed using pre-IFCC calibrated cysC values (Schwartz and Zappitelli). Our cysC values were then divided by the correction factor of 1.17 prior to calculation of eGFR [15].

Equations

1. **Pierce (age and sex dependent):** GFR (ml/min/1.73m^2) $= k * \frac{1}{cysC(mg/l)}$
    - K varies non-monotonically with the lowest value at age 1 (males: k=74.8, females: k=76.5) and the highest value at age 15 for males (k=87.2) and at age 12 for females (k=79.9).
    - PENIA, calibrated to IFCC standards

2. **Pierce (constant):** GFR (ml/min/1.73m^2) $= k * \frac{1}{cysC(mg/l)}$
    - With k=81.9 for males and k=74.9 for females
    - PENIA, calibrated to IFCC standards

3. **Schwartz:** GFR (ml/min/1.73m^2) $= 40.6 * \left(\frac{1.8}{cysC(mg/l)}\right)^{0.93}$
    - PENIA, not calibrated to IFCC standards

4.**Schwartz (extensive equation):**

GFR (ml/min/1.73m^2) = $= 39.1 * [\frac{height\ (m)}{SCr\ (\frac{mg}{dL})}]^{0.516} * \left[\frac{1.8}{CysC\ (\frac{mg}{L})}\right]^{0.294} * \left[\frac{30}{BUN\ (\frac{mg}{dL})}\right]^{0.169} * [1.009]^{male} * \left[\frac{height\ (m)}{1.4}\right]^{0.188}$

- PENIA, not calibrated to IFCC standards

4. **Full Age Spectrum (FAS)$_{\text{cysC}}$:** GFR (ml/min/1.73m^2) $= \frac{107.3}{cysC(mg/l)/0.82}$
    - PENIA, calibrated to IFCC standards

5. **Zappitelli:** GFR (ml/min/1.73m^2) $= \frac{75.94}{cysC(mg/l)^{1.17}}$
    - PENIA, not calibrated to IFCC standards

6. **CAPA:** GFR (ml/min/1.73m^2) = 130 * cysC (mg/L)$^{-1.069}$ * age (years)$^{-0.117}$ -7
    - PENIA, calibrated to IFCC standards
    - For children below the age of 1 years, 1 was used as age term (replacing age (years)$^{-0.117}$)

7. **Pierce (creatinine based):** GFR(ml/min/1.73m^2)= k*height(m)/SCr (mg/dL)

8. **Smeets:** GFR(ml/min/1.73m^2) = 31.0 *height(m)/SCr (mg/dL)

Of note, the Pierce equation was developed for children aged 1 year and older and different k-vales are reported for each year of life, specified for boys and girls. Because no k-values are available for children < 1 year of age and k-values are increasing with increasing age (up to 12 years of age), we used the k-value reported for one-two year olds also for children under the age of one year. This applies both for the cystatin and creatinine based Pierce equation. The Smeets-eGFR equation was only used for neonates up to 28 days of age.

To assess the agreement between several eGFR equations with iohexol-based mGFR, bias and accuracy were calculated. Bias was the median difference between eGFR and mGFR per patient. Comparison of eGFR and mGFR values on a group level was performed using the Wilcoxon signed-rank test for paired data. Accuracy was the percentage of patients having a similar eGFR when compared to mGFR (≤30% difference). Agreement between eGFR and mGFR was visually displayed using Bland-Altman plots and by calculating the limits of agreement. Outliers were not removed from our analysis. In case of missing data, missings were analysed to assess whether these patients were systematically different from the patient included in the analysed cohort. To enable direct comparison to performance of creatinine-based eGFR-equations, we did compare our results to results previously obtained in the same cohort [1]. Statistical analyses were performed using SPSS statistics version 25.0

References (cited in supplement)

1. Smeets NJL, Teunissen EMM, van der Velden K*, et al.* Glomerular filtration rate in critically ill neonates and children: creatinine-based estimations versus iohexol-based measurements. *Pediatric nephrology (Berlin, Germany)* 2022.

2. Tondel C, Bolann B, Salvador CL*, et al.* Iohexol plasma clearance in children: validation of multiple formulas and two-point sampling times. *Pediatric nephrology (Berlin, Germany)* 2017; **32:** 311-320.

3. Hartman SJF, Zwiers AJM, van de Water NEC*, et al.* Proenkephalin as a new biomarker for pediatric acute kidney injury - reference values and performance in children under one year of age. *Clinical chemistry and laboratory medicine* 2020; **58:** 1911-1919.

4. Zwart TC, de Vries APJ, Engbers AGJ*, et al.* Model-Based Estimation of Iohexol Plasma Clearance for Pragmatic Renal Function Determination in the Renal Transplantation Setting. *Clinical pharmacokinetics* 2021; **60:** 1201-1215.

5. Committee for Medicinal Products for Human Use (CHMP) EMA. Guideline on bioanalytical method validation. 2011.

6. Jodal L, Brochner-Mortensen J. Reassessment of a classical single injection 51Cr-EDTA clearance method for determination of renal function in children and adults. Part I: Analytically correct relationship between total and one-pool clearance. *Scandinavian journal of clinical and laboratory investigation* 2009; **69:** 305-313.

7. Haycock GB, Schwartz GJ, Wisotsky DH. Geometric method for measuring body surface area: a height-weight formula validated in infants, children, and adults. *The Journal of pediatrics* 1978; **93:** 62-66.

8. van der Sijs H, Guchelaar HJ. Formulas for calculating body surface area. *Ann Pharmacother* 2002; **36:** 345-346.

9. Pierce CB, Muñoz A, Ng DK*, et al.* Age- and sex-dependent clinical equations to estimate glomerular filtration rates in children and young adults with chronic kidney disease. *Kidney international* 2020**:** 948-956.

10. Schwartz GJ, Schneider MF, Maier PS*, et al.* Improved equations estimating GFR in children with chronic kidney disease using an immunonephelometric determination of cystatin C. *Kidney international* 2012; **82:** 445-453.

11. Pottel H, Delanaye P, Schaeffner E*, et al.* Estimating glomerular filtration rate for the full age spectrum from serum creatinine and cystatin C. *Nephrol Dial Transplant* 2017; **32:** 497-507.

12. Zappitelli M, Parvex P, Joseph L*, et al.* Derivation and validation of cystatin C-based prediction equations for GFR in children. *American journal of kidney diseases : the official journal of the National Kidney Foundation* 2006; **48:** 221-230.

13. Grubb A, Horio M, Hansson LO*, et al.* Generation of a new cystatin C-based estimating equation for glomerular filtration rate by use of 7 assays standardized to the international calibrator. *Clin Chem* 2014; **60:** 974-986.

14. Schwartz GJ, Munoz A, Schneider MF*, et al.* New equations to estimate GFR in children with CKD. *Journal of the American Society of Nephrology : JASN* 2009; **20:** 629-637.

15. Schwartz GJ, Cox C, Seegmiller JC*, et al.* Recalibration of cystatin C using standardized material in Siemens nephelometers. *Pediatric nephrology (Berlin, Germany)* 2020; **35:** 279-285.

Supplemental references (cited in main manuscript)

S1. Hidayati EL, Utami MD, Rohsiswatmo R, *et al.* Cystatin C compared to serum creatinine as a marker of acute kidney injury in critically ill neonates. *Pediatric nephrology (Berlin, Germany)* 2021; **36**:181-6.

S2. Herrero-Morín JD, Málaga S, Fernández N, *et al.* Cystatin C and beta2-microglobulin: markers of glomerular filtration in critically ill children. *Critical care (London, England).* 2007; **11**: R59.

S3. Ataei N, Bazargani B, Ameli S, *et al.* Early detection of acute kidney injury by serum cystatin C in critically ill children. *Pediatric nephrology (Berlin, Germany).* 2014; **29**: 133-8.

S4. Smeets N, IntHout J, van der Burgh M, *et al.* Maturation of Glomerular Filtration Rate in Term-Born Neonates: An Individual Participant Data Meta-Analysis. *Journal of the American Society of Nephrology: JASN.* 2022; **7**: 1277-1292

S5. Zwiers AJ, Cransberg K, de Rijke YB, *et al.* Reference ranges for serum β-trace protein in neonates and children younger than 1 year of age. *Clinical chemistry and laboratory medicine.* 2014; **52**: 1815-21.

S6. Mussap M, Plebani M. Biochemistry and clinical role of human cystatin C. *Crit Rev Clin Lab Sci.* 2004; **41:** 467-550.
